# Validation of the National Institutes of Health Stroke Scale in Intracerebral Hemorrhage

**DOI:** 10.1161/SVIN.123.000834

**Published:** 2023-06-09

**Authors:** Wendy Dusenbury, Georgios Tsivgoulis, Jason Chang, Nitin Goyal, Victoria Swatzell, Andrei V. Alexandrov, Patrick Lyden, Anne W. Alexandrov

**Affiliations:** ^1^ University of Tennessee Health Science Center Memphis TN; ^2^ University of Athens Athens Greece; ^3^ Washington Hospital Center Washington DC; ^4^ Banner University Hospital University of Arizona Phoenix AZ; ^5^ Zilkha Neurogenetic Institute at the Keck School of Medicine of USC Los Angeles CA

**Keywords:** Glasgow coma scale, intracerebral hemorrhage, NIH stroke scale

## Abstract

**Background:**

We sought to determine if the National Institutes of Health Stroke Scale (NIHSS) has a greater discriminative power than Glasgow coma scale (GCS) to identify patients at risk of poor early functional outcomes and large hematoma volumes.

**Methods:**

We prospectively collected clinical assessments, imaging, and outcome data in consecutive patients with intracerebral hemorrhage, and determined the ability of GCS and NIHSS to predict poor functional outcome (modified Rankin scale 3–6) and hematoma volume >30 cm^3^ using receiver operating characteristics analysis, C‐statistics, and the DeLong test.

**Results:**

We studied 672 patients with intracerebral hemorrhage (mean age 62±14 years; 56% men; median intracerebral hemorrhage score=1, interquartile range (IQR) 0–2; median intracerebral hemorrhage volume 7 cm^3^, IQR 2–19) with median NIHSS of 8 (IQR 3–18) and GCS 15 (IQR 7–15). NIHSS correlated strongly to GCS (r=−0.773; *P*<0.001). Admission NIHSS (C‐statistic: 0.91; 95% CI, 0.89–0.93) predicted better than GCS (0.78; 95% CI, 0.75–0.81) discharge poor functional outcome (DeLong test *P*<0.001). NIHSS (0.82; 95% CI, 0.78–0.86) also discriminated better than GCS (0.78; 95% CI, 0.73–0.83) patients with large hematoma volume (DeLong test *P*=0.029).

**Conclusion:**

The NIHSS has a greater discriminative power than GCS to identify patients at risk of poor early functional outcomes and large hematoma volumes.

## Graphical abstract

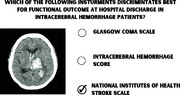

**Nonstandard Abbreviations and Acronyms**

GCS: Glasgow coma scale
ICH: intracerebral hemorrhage
LOC: level of consciousness
mRS: modified Rankin scale
NIHSS: National Institutes of Health Stroke Scale

**Clinical Perspective**

- The National Institutes of Health Stroke Scale score discriminates better than the Glasgow Coma Score for poor discharge modified Rankin scale (scores 3–6) outcomes and large hematoma volumes (>30 cm^3^).
- In patients with acute intracerebral hemorrhage, every 1‐point increase in the National Institutes of Health Stroke Scale score increases the odds of poor discharge modified Rankin scale (3–6) by 1.28; excluding modified Rankin scale 6, the odds of inability to walk independently at hospital discharge increase by 1.61 with every 1‐point increase in the National Institutes of Health Stroke Scale score.
- The National Institutes of Health Stroke Scale should be used for both initial and serial clinical assessment of patients with intracerebral hemorrhage to document focal neurologic disability indicative of improvement or deterioration throughout the hospital admission.

Intracerebral hemorrhage (ICH) is a catastrophic event associated with high mortality and morbidity,^1^ carrying a fatality rate of approximately 40% at 1 month and 54% at 1 year.^2^ Early hematoma expansion occurs in up to 40% of patients with ICH^3^, ^4^, ^5^ being independently associated with early neurological deterioration,^6^, ^7^ poor functional outcome, and death.^8^, ^9^ Only 12%–39% of surviving patients with ICH ever achieve long‐term functional independence.^2^ Despite the devastating nature of this disease, debate remains as to what neurologic assessment tool would best support the initial examination for early prognostication.

The ICH score^10^ was developed as a prognostic tool using predictors of poor clinical outcome such as hematoma volume, location, patient age, and level of consciousness (LOC). However, the ICH score itself lacks utility as a measure of neurologic disability. Clinical assessment in ICH is most commonly done with the Glasgow coma scale (GCS),^11^ but as an LOC tool, the GCS also lacks details of focal neurologic findings. The National Institutes of Health Stroke Scale (NIHSS)^12^ has been validated as a measure of ischemic stroke severity^13^ and more recently has been coopted as an ischemic stroke outcome predictor.^14^, ^15^, ^16^, ^17^ Use of the NIHSS in patients with ICH may provide a more detailed picture of stroke severity and neurological disability than the GCS alone.^18^, ^19^ Therefore, we sought to examine the utility of the admission NIHSS in comparison to the GCS to discriminate early poor functional outcome at discharge and hematoma volume in a prospective cohort of patients with ICH.

## Methods

Parties interested in the data from this study should contact the primary investigator who will work with the Institutional Review Board to make deidentified data available.

Institutional Review Board approval was obtained for prospective collection of hospital data on patients with acute ICH admitted to a tertiary care comprehensive stroke center in the Mid‐south region of the United States stroke belt. We included consecutive adult (≥18 years of age) patients over a 5‐year period with spontaneous ICH with last known well within 24 hours of comprehensive stroke center hospital admission and excluded patients with ICH due to trauma, brain tumors, venous sinus thrombosis, and ICH from underlying structural vascular lesions. All ICHs were initially admitted to the neurointensive care unit. As per hospital protocol, patients were treated with intravenous antihypertensive agents to reach a goal systolic blood pressure <140 mmHg during the first 24 hours after admission. Systolic blood pressure goal <160 mmHg was used in patients with admission systolic blood pressure >220 mmHg and acute kidney impairment.^20^ Stability head computed topography scans for hemorrhage volume were obtained at 6 hours postadmission as standard of care.

Deidentified patient data were collected prospectively including both GCS and NIHSS assessments by certified investigators, alongside demographics including race and ethnicity as described by patients (or family member) to the hospital admissions clerk, discharge modified Rankin scale (mRS), neuroimaging findings including calculation of hematoma volume, presence of intraventricular hemorrhage, bleed location, and hospital discharge outcomes. Hematoma volume was calculated using the (AxBxC)/2 method^21^ and bleeds greater than 30 cm^3^ were classified as large hematoma volume.^22^ mRS scores at discharge were dichotomized to good functional outcome (mRS 0—2) and poor functional outcome (mRS 3—6).

Data were entered and analyzed with Statistical Package for the Social Sciences (IBM version 25). Data are presented as mean±SD or median with interquartile range (IQR, 25th–75th percentile), if not normally distributed. We evaluated the predictive ability of NIHSS and GCS score for the detection of poor functional outcome at discharge and large hematoma volumes using receiver operating characteristic curve models. Areas under receiver‐operator curves (c statistic) and corresponding 95% CIs were calculated as a measure of predictive ability. The C‐statistic integrates sensitivity and specificity of the range of a variable and estimates how well a prediction rule can correctly rank‐order patients by risk. Ideal prediction produces a C‐statistic of 1.00; prediction no better than chance is associated with a C‐statistic of ≤0.50. The C‐statistics of NIHSS and GCS scores for predicting poor functional outcome at discharge and large hematoma volumes were compared using DeLong test.^23^ The correlation of NIHSS and GCS scores was evaluated by Spearman correlation.

## Results

Between January 2011 and December 2015, we identified 672 patients with ICH in our prospective registry: mean age 62±14 years; 56% men, 58% Black, 41% White, and 22% were of Latino ethnicity (Table[Table svi212759-tbl-0001]). Common comorbidities included hypertension (86%), diabetes (35%), active cigarette smoking (34%), and hyperlipidemia (32%), with 26% taking statin medications, 32% on antiplatelet medications, and 4% on anticoagulant medication.

**Table svi212759-tbl-0001:** Sample Characteristics

Characteristics	Results
Sex	
Male	56%
Female	44%
Age	62±14 (median 60 [IQR 52–72]) years
Race	
White	275 (41%)
Black	388 (58%)
Asian	5 (0.5%)
Other*	4 (0.5%)
Latino ethnicity	22%
Hypertension	86%
Active smoker	34%
Diabetes	35%
Admission hemoglobin A1c	6.2±1.7 (median 5.7 [IQR 5.3–6.4]) %
Hyperlipidemia	32%
Statin medication	26%
Antiplatelet medication	32%
Anticoagulation medication	4%
Admission INR	1.1±0.37 (median 1.0 [IQR 1.0–1.1])
Body mass index	28.9±7.4 (median 27.5 [23.7–32.6]) kg/m^2^
Location of ICH	
Basal ganglia	34%
Thalamus	19%
Brainstem	6%
Cerebellum	8%
Lobar	30%
Centrum semiovale	1%
Other	2%
Admission volume of ICH	14.2±18.1 (median 7.0 [IQR 2.3–18.4]) cm^3^
Intraventricular hemorrhage	45%
24 h hematoma expansion	17%
ICH score	Median 1 (IQR 0–2)
Admission GCS score	Median 15 (IQR 7–15)
Admission NIHSS score	Median 8 (IQR 2–18)
mRS score at discharge	Median 4 (IQR 2–5)
mRS 3–6 at discharge	67.5%
Length of stay	10.1±11.2 (median 6 [IQR 3–12]) days
Discharge disposition:	
Home	30%
Inpatient rehabilitation	27%
Skilled nursing/long‐term care	16%
Hospice	3%
Died	24%

GCS indicates Glasgow coma scale; ICH, intracerebral hemorrhage; IQR, interquartile range; mRS, modified Rankin scale; and NIHSS, National Institutes of Health Stroke Scale.

^*^ Other represents racial categories not collected in the hospital admissions database, including mixed races.

Overall, median ICH score for the cohort was 1 (IQR 0—2) with a median ICH volume of 7 cm^3^ (IQR 2—18 cm^3^) on admission computed topography. The majority (34%) of hemorrhages were in the basal ganglia and 14% were infratentorial. Forty‐five percent of patients had intraventricular extension and 17% of patients had hemorrhage volume expansion on 6‐hour postadmission stability computed topography scans or repeat computed topography scans for clinical deterioration within the first 24 hours of ictus.

Median admission NIHSS was 8 points (IQR 2–18), and median admission GCS was 15 points (IQR 7–15). Median discharge mRS was 4 (IQR 2–5), with 67.5% of the sample having mRS‐scores of 3–6 and overall, 24% of the sample dying during hospitalization. The admission NIHSS showed a strong negative correlation to the admission GCS (r=−0.773; *P*<0.001).

The admission NIHSS (C‐statistic: 0.91; 95% CI: 35 0.89–0.93) discriminated better than admission GCS (C‐statistic: 0.78; 95% CI: 0.75–0.81) for poor discharge mRS (DeLong test *P*<0.001; Figure[Fig svi212759-fig-0001]). Given the large number of deaths (24%) in the cohort, we also explored discrimination of the NIHSS and GCS for discharge mRS 3–5 points, finding that the admission NIHSS (C‐statistic: 0.87; 95% CI: 0.845–0.90) also discriminated better than the admission GCS (C‐statistic: 0.68; 95% CI: 0.64–0.73) for poor discharge functional outcome. The admission NIHSS (C‐statistic: 0.82; 95% CI: 0.78–0.86) also discriminated better than GCS (C‐statistic: 0.78; 95% CI: 0.73–0.83) for large hematoma volume (DeLong test *P*=0.029; Figure[Fig svi212759-fig-0001]).

**Figure svi212759-fig-0001:**
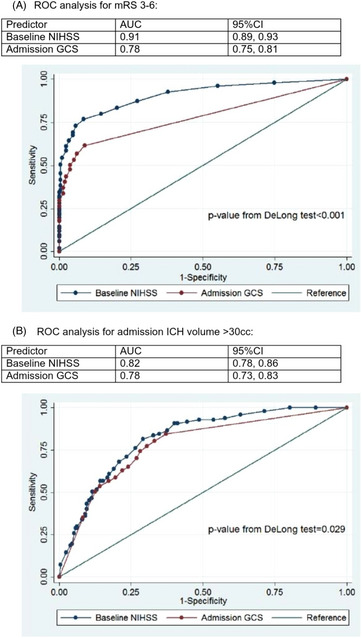
Receiver operating curve analysis **A**, Receiver operating curve analysis of admission National Institutes of Health Stroke Scale (NIHSS) and the admission Glasgow coma scale (GCS) for modified Rankin scales (mRS) at discharge from intracerebral hemorrhage ictus. **B**, Receiver operating curve analysis of the admission NIHSS and the admission GCS for intracerebral hematoma volume.

Thirty percent of patients were discharged home. For each point increase in the NIHSS, the odds of a discharge mRS 3–6 increased by a factor of 1.28 (95% CI,1.23–1.34; *P*<0.001). Excluding those patients who died in hospital, for every point increase in the NIHSS, the odds of not being able to independently walk at the time of discharge increased by a factor of 1.61 (95% CI,1.48–1.74; *P*<0.001).

## Discussion

We believe that this is the largest prospectively collected ICH data set in which the NIHSS was obtained and contrasted with ICH volume and functional status at discharge. Our findings indicate that the admission NIHSS is a better discriminator for large admission hematoma volume and poor functional status at discharge compared with the GCS. Our findings are consistent with others showing that the NIHSS correlated strongly with hematoma volume,^24^, ^25^, ^26^ and hematoma volume is considered an important predictor of ICH outcome.^26^ The mechanism of the ability of the NIHSS to discriminate hematoma volume better than the GCS is not clear, but may result from the NIHSS scale design: the scale contains 2 main factors corresponding to right or left hemisphere.^27^ Because hematoma expansion is often associated with neurological deterioration and poor clinical outcome,^8^, ^28^ the ability to capture neurological deterioration beyond LOC would benefit patient management, especially in the early hours following ICH ictus when hemorrhage expansion is most likely. While the ICH Score is a widely used scale that has been externally validated for outcome prognostication,^29^ its usefulness is limited due to unsuitability for serial assessments^30^ and poor discrimination of the spectrum of functional outcomes beyond mortality.

The GCS was developed in 1974 as an assessment of the “depth” of impaired consciousness in patients with acute cerebral disorders.^31^ As an LOC measure, use of the GCS has been favored historically for serial assessments in ICH^26^, ^32^ since LOC (inversely related to hematoma volume) is a key determinant of outcome. However, the GCS fails to provide the variety of clinically important focal assessment data contained within the NIHSS. In our study median GCS was 15 (normal) since most patients were awake on admission yet had sizeable neurologic deficits. This limitation of the GCS has been noted previously in a substantial number of patients with acute stroke with measurable disabling deficits on the NIHSS and no deficit on GCS.^30^

Neurologic assessments are essential to understanding stability, improvement, and deterioration in patients with acute stroke,^33^ and while both GCS and NIHSS are used in patients with ICH^34^, ^35^ consensus on the use of a specific scale for baseline and serial assessment in the patient with ICH has been lacking.^30^ However, the admission NIHSS has been found to correlate with functional outcomes after stroke.^24^, ^33^, ^36^, ^37^ Decreased LOC on presentation has been thought to diminish the utility of the NIHSS^29^; however, ICH mortality has been found to be predicted better with the NIHSS than GCS.^38^

Our work has limitations, including use of a single site for this study with sampling obtained within 1 geographical region. We also recognize that there is subjectivity within the NIHSS itself and varying levels of competency between examiners could potentially cause discrepancies in scoring^39^; however, all NIHSS scores in our study were obtained by highly experienced stroke and neurocritical care physicians and nurse practitioners who were all certified^40^ in use of the NIHSS. Another limitation is that our work does not exclude patients undergoing early withdrawal in care; however, given the aggressive nature of our stroke and neurocritical teams’ approach to ICH resuscitation it is unlikely that a bias toward early withdrawal of care was a major contributor to the death rate in our sample. Lastly, our work is limited to findings obtained during the early hospitalization period when ICH worsening is most common, and as such, it does not address longitudinal morbidity and mortality in our subjects. Despite this limitation, previous work has shown early NIHSS scores are predictive of 30‐day and 5‐year mortality, as well as long term functional outcome in survivors.^36^

In conclusion, our study supports the use of NIHSS for the initial assessment of acute patients with ICH and this information can provide baseline measurement of prognostic value to identify patients at risk of devastating neurologic disability due to variety of contributing factors since ICH is a dynamic process.^5^, ^41^ While our study did not evaluate use of the NIHSS as a serial measure in ICH, future work should explore this as the NIHSS may improve early recognition of neurological deterioration without LOC decrease as well as mounting disability over time, enabling better prognostication and earlier implementation of injury‐reducing strategies.

## Sources of Funding

None.

## Disclosures

None.
